# The Oleaginous Yeast *Meyerozyma guilliermondii* BI281A as a New Potential Biodiesel Feedstock: Selection and Lipid Production Optimization

**DOI:** 10.3389/fmicb.2017.01776

**Published:** 2017-09-22

**Authors:** Mauricio Ramírez-Castrillón, Victoria P. Jaramillo-Garcia, Priscila D. Rosa, Melissa F. Landell, Duong Vu, Mariana F. Fabricio, Marco A. Z. Ayub, Vincent Robert, João A. P. Henriques, Patricia Valente

**Affiliations:** ^1^Graduate Program in Cell and Molecular Biology, Biotechnology Center, Federal University of Rio Grande do Sul Porto Alegre, Brazil; ^2^Department of Microbiology, Immunology and Parasitology, Federal University of Rio Grande do Sul Porto Alegre, Brazil; ^3^Research Group in Mycology (GIM), Research Center in Environmental Basic Sciences (CICBA), Faculty of Basic Sciences, Universidad Santiago de Cali Cali, Colombia; ^4^Graduate Program in Medical Sciences, Federal University of Rio Grande do Sul Porto Alegre, Brazil; ^5^Genetics Section/ICBS, Federal University of Alagoas Maceió, Brazil; ^6^Bioinformatics Research Group, Westerdijk Fungal Biodiversity Institute Utrecht, Netherlands; ^7^Biotechnology, Bioprocess, and Biocatalysis Group, Food Science and Technology Institute, Federal University of Rio Grande do Sul Porto Alegre, Brazil

**Keywords:** biodiesel, fatty acids, raw glycerol, lipid production, Nile Red

## Abstract

A high throughput screening (HTS) methodology for evaluation of cellular lipid content based on Nile red fluorescence reads using black background 96-wells test plates and a plate reader equipment allowed the rapid intracellular lipid estimation of strains from a Brazilian phylloplane yeast collection. A new oleaginous yeast, *Meyerozyma guilliermondii* BI281A, was selected, for which the gravimetric determination of total lipids relative to dry weight was 52.38% for glucose or 34.97% for pure glycerol. The lipid production was optimized obtaining 108 mg/L of neutral lipids using pure glycerol as carbon source, and the strain proved capable of accumulating oil using raw glycerol from a biodiesel refinery. The lipid profile showed monounsaturated fatty acids (MUFA) varying between 56 or 74% in pure or raw glycerol, respectively. *M. guilliermondii* BI281A bears potential as a new biodiesel feedstock.

## Introduction

Biodiesel production employing oleaginous microorganisms is a promising alternative to overcome the critical bottlenecks of the first-generation biodiesel (Sitepu et al., 2014), which uses vegetable oil as raw material and competes with human food. Yeasts are a promising source of microbial oil (Poli et al., 2013, 2014b; Sitepu et al., 2014), since these microorganisms can accumulate up to 70% of their dry weight as lipids (Angerbauer et al., 2008). Although several oleaginous yeasts have been described in literature as potential producers of oil as raw material for biodiesel (Ageitos et al., 2011; Duarte et al., 2013; Poli et al., 2013, 2014a; Garay et al., 2016), biofuel production from yeast oil is not yet a reality, mainly due to the high cost of microbial oil production. Therefore, efforts are necessary to assess new lipid-producing yeasts and to enhance their lipid production using low cost raw materials to turn yeast oil applications cost-effective.

In the light of these considerations, the main objective of this study was to select a new oleaginous yeast strain from a Brazilian culture collection, capable of accumulating oil using raw glycerol, a sub-product of biodiesel refineries, as carbon source. A high throughput screening (HTS) methodology permitted the comprehensive evaluation of the lipid-accumulating ability of yeast strains and the selection of a new oleaginous yeast, identified as *Meyerozyma guilliermondii* BI281A. Finally, culture conditions were optimized for improving its lipid production.

## Methods

### Microorganisms

The yeast strains tested were recovered from a yeast culture collection located at Federal University of Rio Grande do Sul (Porto Alegre, Brazil, Table S1). We used *Y. lipolytica* QU21 as a reference strain of oleaginous yeast (Poli et al., 2013). *S. cerevisiae* CBS 1171 was used as negative control in the experiments. *M. guilliermondii* BI281A was identified using molecular sequencing of ITS region, according to Ramirez-Castrillon et al. (2014) (Genbank accession number: MF599409). The strain was deposited at the Collection of Microorganisms, DNA and Cells of Federal University of Minas Gerais (UFMG) under the code CM-UFMG-Y6124.

### Media

GYP medium (10 g/L yeast extract, 10 g/L peptone, 20 g/L glucose) or YM medium (3 g/L yeast extract, 3 g/L malt extract, 10 g/L peptone, and 20 g/L glucose) were used for pre-cultivation of the strains. Media A, A-glycerol, and A-raw glycerol had the following composition: 1 g/L KH_2_PO_4_, 1 g/L (NH_4_)_2_SO_4_, 0.5 g/L MgCl2-6H_2_O, plus 100 g/L glucose, 15% (v/v) glycerol, or 15% (v/v) raw glycerol, respectively. Raw glycerol was supplied by an oil-based biodiesel factory (Canoas, Brazil), and contains: (as weight percentage) 82.97% glycerol, 10.62% moisture, 5.72% NaCl, 0.75% mono-glycerides, and trace-concentration of ashes and residual methanol.

### High throughput screening (HTS) methodology

The HTS methodology was based on Sitepu et al. (2012) with modifications. The strains were initially grown in GYP or YM medium for 48 h at 28°C to obtain metabolically active cells. Afterwards, each strain was transferred to 25 mL of medium A (glucose) in a 125 mL flask and grown for 24 h at 28°C and 150 rpm. 1 mL of the pre-culture (7 × 10^7^ cells/mL) was inoculated in 75 mL of A medium in a 250 mL flask (to maintain a ratio of 2/3 as free volume as mentioned by Poli et al., 2014b) for 72 h, 28°C and 150 rpm on shaker.

A volume of 2 mL of culture was centrifuged (4,293 g for 5 min) and the cell pellet was resuspended in a solution of PBS 1X (137 mM NaCl, 2.7 mM KCl, 8 mM Na_2_HPO_4_, and 2 mM KH_2_PO_4_), isopropanol 5% (v/v) and Tween 20 in a Critical Micelle Concentration (CMC 0.06 mM). An aliquot of 150 μL (10^7^ cells/mL) was transferred to a black background 96-wells test plate (Jet Biofil, China) and the relative fluorescence was measured in a Perkin Elmer Enspire Multimode Plate Reader 2300 equipment (488 nm of excitation, 585 nm of emission). After measuring the basal fluorescence intensity in each sample without dye, we added 50 μL of Nile Red (50 mg/L) to the reaction, incubated inside the equipment for 10 min with shaking, and finally measured the samples with dye. The differences in readings of the samples with dye, without dye, and the blank (without sample) were the relative fluorescence expressed as RFU (Relative Fluorescence Units). Each sample had technical and biological triplicates. We used *Y. lipolytica* QU21 as positive control, and *S. cerevisiae* CBS 1171 as negative control. We considered strains with RFU equal or higher than QU21 as potential oleaginous yeasts.

### Calibration curve

We constructed a calibration curve to obtain an estimation of the lipid content within yeast cells using a triolein solution ($C18:1_{3}^{\Delta 9}$, Sigma Aldrich), in the range of 10–50 mg/L dissolved in PBS 1X, isopropanol 5% (v/v), and Tween 20 in CMC (0.06 mM). The lipid content was estimated in comparison with the calibration curve and expressed as mg lipids/ L medium.

### Gravimetric determination of biomass

For gravimetric analysis of biomass, we transferred 45 mL of the grown cultures to 50 mL conical tubes, and centrifuged at 4,233 g for 10 min to remove the supernatant, followed by two washings using 15 mL of PBS 1X each time. We dried the cell pellets at 60°C until constant weight. We measured the biomass using an analytical balance (Shimadzu AY220), and the dry cells were used for lipid extraction. All experiments were performed in triplicate.

### Gravimetric determination of total lipids

We extracted the lipids from the biomass following the method of Bligh and Dyer (1959). Dry biomass was suspended in chloroform/methanol (2:1, v/v) and cell lysis occurred using a Turrax homogenizer (ULTRA-TURRAX T18; IKA), with four cycles of homogenizing for 2 min and ice cooling for 1 min to avoid lipid heating. For determination of the total lipid content, solvents were evaporated at 60°C using a rotatory evaporator (Laborota 4,000eco) and dried at 60°C for 24h. Finally, extracted lipid weight was measured using an analytical balance (Shimadzu AY220). We expressed the lipid yield as concentration (g/L medium), the lipid content as a percentage of the lipid weight relative to dry biomass weight (% w/w) and the productivity as g lipid/(g biomass × h).

### Lipid profile

We transesterified the dried lipid extracts following the method of Hartman and Lago (1973). A volume of 1 mL of fatty acid methyl esters (FAME) was transferred to a 2 mL GC vial insert, which contained 0.5 mL of BHT (butylated hydroxytoluene) solution (0.4 mg/mL). The FAME profiling and quantification were performed using a gas chromatograph (GC-2010 PLUS, SHIMADZU) fitted with a split injector and a flame ionization detector (FID) with hydrogen as carrier gas with a flow of 57.1 mL/min, equipped with a SUPELCO SLB-IL100 Capillary GC Column (30 m length, internal diameter of 0.25 mm with fused-silica column coated with a 0.2 μm polyethylene glycol film) (Cat. # 28884-U, Sigma-Aldrich). Samples of 1.0 μL were injected using a 10.0 μL syringe. The detector temperature was set to 250°C; the carrier gas was hydrogen at a flow-rate of 40 mL/min, an average velocity of 31 cm/s, and a constant pressure of 46.0 kPa. The airflow was 400 mL/min. Initial temperature was 50°C, increasing to 240°C at 3°C/min, held for 10 min at 240°C.

We processed the peak areas of the fatty acids using GC solution Postrun (version 2.42 SU1, Shimadzu), and determined the fatty acid methyl ester profile of each sample by comparing the retention time of each FAME peak present in the sample with the retention time of FAME reference standards contained in the reference standard mixture SUPELCO SLB-IL100 Capillary GC Column. We included BHT in the GC vial insert for each sample as the internal standard for quantification of fatty acids. We calculated the percentage of total fatty acid content as the ratio of individual FAME peak area to the sum of the all FAME peak areas, excluding the BHT peak.

### Optimization of lipid production

We assessed four variables independently to improve the lipid production in *M. guilliermondii* BI281A: carbon source (glucose or glycerol), nitrogen source [malt extract, peptone, tryptone, NH_4_NO_3_, and (NH_4_)_2_SO_4_], time of cultivation (24–120 h) and temperature (20, 26, 28, 30, and 37°C). To evaluate the carbon and nitrogen sources, we modified medium A to change the default carbon or nitrogen component in the preparation, respectively. For example, we changed 100 g/L of glucose by 15% (v/v) of glycerol. The response variables were dry biomass (g/L), optical density (OD_600 nm_), and lipid production (mg/L estimation from Relative Fluorescence). All experiments were measured in technical and biological triplicates.

Based on the univariate results, we chose three independent variables for the optimization of lipid production with glycerol and ammonium sulfate as carbon and nitrogen sources, respectively, using Central Composite Design (CCD), and Response Surface Methodology (RSM). The dependent variable selected for this study was lipid production (mg/L). The independent variables were cultivation time (X_1_), medium C/N ratio (X_2_), and stirring (rpm) (X_3_). Each variable was studied in three levels (−α, 0, +α) with two axial points (−1.68 α and +1.68 α), and the range and levels of these variables are shown in Table 1. The experimental design included 15 runs with three replicates in the central point. The mathematical relationship between the response variable (lipid production) and variables X1, X2, X3 was approximated by a quadratic model equation, according to the general second-degree polynomial Equation (1):

(1) $$\text{Z}=\;\beta_{0}+\sum \beta_{\text{i}}{\text{X}}_{\text{i}}+\sum \beta_{\text{ij}}{\text{X}}_{\text{i}}{\text{Y}}_{\text{j}}+\sum \beta_{\text{ii}}{{\text{X}}_{\text{i}}}^{2}$$

where Z is the predicted response (lipid production, mg/L); β_0_ is the intercept, β_i_ the linear coefficient, β_ij_ the quadratic coefficient, β_ii_ is the linear interaction between X_i_ and Y_j_ regression coefficients, and X_i_, Y_j_ are the assessed variables (X_1_, X_2_, X_3_) that define the mathematical function of *Z*. The precision of the above polynomial model was evaluated by the coefficient of determination R^2^, and the statistical significance was determined by the *F*-test.

**Table 1:** Coded values of independent variables at different levels used in Central Composite Design.

**Independent variable**	**Symbol**	**Level**				
		**−1.68α**	**−α**	**0**	**+α**	**+1.68α**
Time (hours)	X3	36	48	72	96	120
C/N ratio^*^	X2	16:1	50:1	100:1	150:1	184:1
Stirring (rpm)	X1	0	50	150	225	276

* *Glycerol/Ammonium sulfate ratio in the A-Glycerol medium*.

### Statistical analysis

Statistical analyses of differences between the relative fluorescence units in the screening experiments were evaluated with paired t-student tests compared to positive or negative controls (*P* < 0.05). We compared carbon and nitrogen sources, and temperatures in the univariate tests with analysis of variance (ANOVA) and uncorrected Fishers LSD test (*P* < 0.05). The calibration curve was determined with a linear regression between relative fluorescence units and triolein concentration. Multivariate analysis was performed with a multivariate analysis of variance (MANOVA, *P* = 0.05). We performed a Chi-square test to compare lipid production in the optimal point with the predicted value given by the model. We performed all statistical analyses using the STATISTICA 13 software package (Statsoft, Dell, USA) and plotted the graphics using Prism 7 (GraphPad Software Inc, USA).

## Results

### The use of HTS methodology for oleaginous yeast screening and the selection of *M. guilliermondii* BI281A

**Figure 1:**
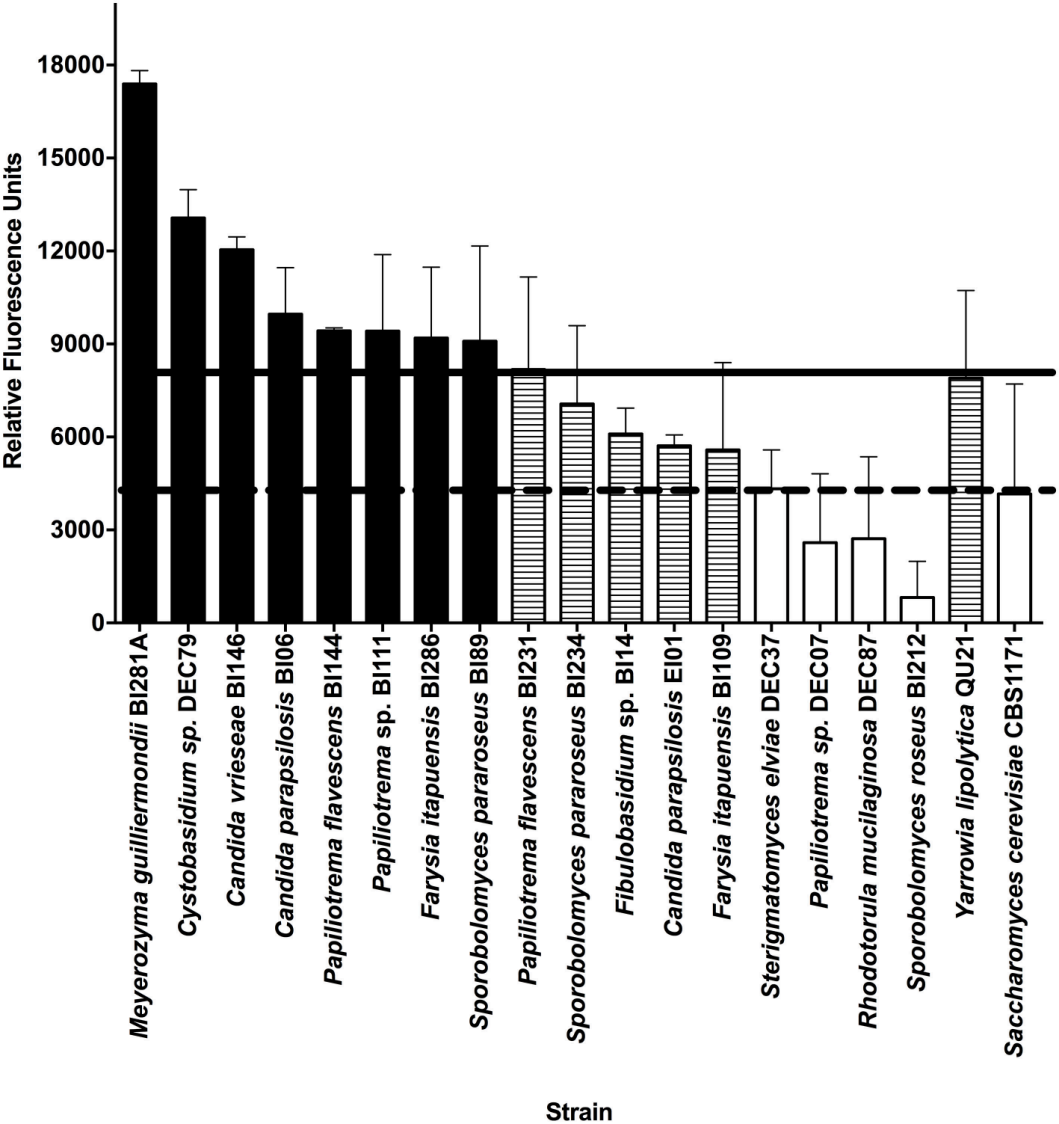
Fluorescence intensity of yeast strains grown in medium A using Nile Red dye (50 mg mL^−1^). Absorption and Emission wavelengths were 488 and 585 nm, respectively. The horizontal solid line indicates the average Relative Fluorescence Units (RFU) of the positive control (*Y. lipolytica* QU21) and the horizontal dotted line indicates the average RFU of the negative control (*S. cerevisiae* CBS 1171). Strains with RFU above the positive control are black-filled, strains between positive control and negative control are pattern-filled and strains with RFU below the negative control are not filled. Strains without fluorescence emission were not included in the figure. All experiments were done in biological and technical triplicates.

A total of 43 yeast strains were screened for lipid production (Table S1). We established two criteria to search for a candidate oleaginous yeast: (1) exponential growth until 72 h and (2) fluorescence intensity in the HTS methodology higher than our positive control (*Y. lipolytica* QU21). Many of the strains assessed showed a delayed growth (19 isolates, sometimes one/two weeks), and were not further tested. After selecting the yeast group with exponential growth up to 72 h, we measured the fluorescence intensity using Nile Red dye according to the described HTS methodology to assess the lipid content of cells. We compared the average (technical and biological triplicates) RFU of yeast strains with the average RFU of the positive control (*Y. lipolytica* QU21; black horizontal line) and the negative control (*S. cerevisiae* CBS 1171; red horizontal line) using A-medium for cell growth (Figure 1). *M. guilliermondii* BI281A and *Cystobasidium* sp. DEC79 strains showed higher RFU than *Y. lipolytica* QU21.

To estimate the lipid production, we initially correlated a calibration curve of triolein concentrations with the fluorescence intensity with Nile Red, according to Poli et al. (2014a), showing a strong correlation between the variables (*R*^2^ = 0.9477, *P* = 0.001). Using this calibration curve, the measurements of fluorescence intensity for strains BI281A, DEC79, and QU21 (positive control) were transformed in lipid production (mg/L) (Figure S1). This result suggested that *M. guilliermondii* BI281A and *Cystobasidium* sp. DEC79 are candidate oleaginous yeasts, but only BI281A showed higher lipid production than QU21 (*P* < 0.05). For this reason, we selected BI281A to optimize the culture media.

### Optimization of conditions to increase lipid production in *M. guilliermondii* BI281A

**Figure 2:**
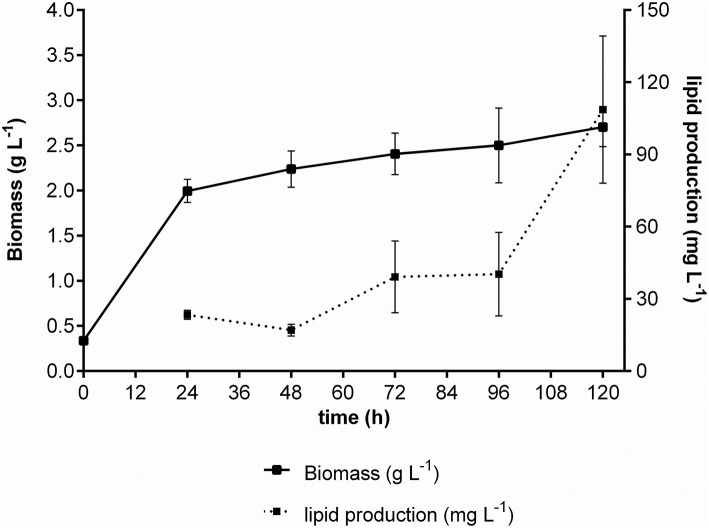
Growth curve of *M. guilliermondii* BI281A using glucose as carbon source. The solid line represents dry biomass (g/L) and the dotted line represents the lipid production (mg/L). All experiments were done in biological triplicate.

**Figure 3:**
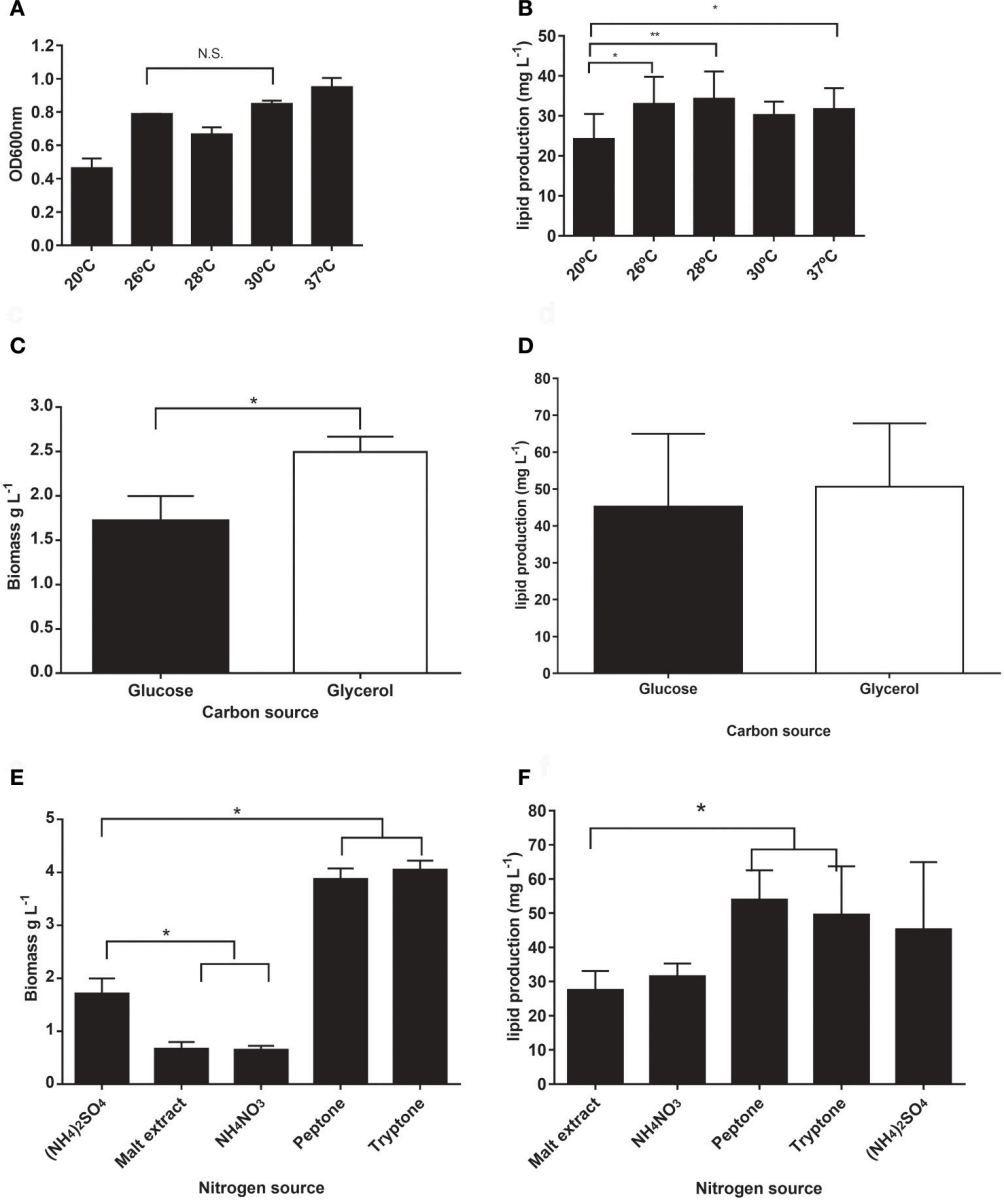
**(A,B)** Comparison of different temperatures in optical density (OD600 nm) and lipid production (mg/L) of *M. guilliermondii* BI281A. **(C,D)** Assessment of glucose and glycerol as carbon source, and **(E,F)** different nitrogen sources in *M. guilliermondii* BI281A biomass (g/L) or lipid (mg/L) production after 72 h of cell growth, 26°C, 150 rpm (medium A). All experiments were assessed independently by biological and technical triplicates. ^*^P < 0.05, ^**^P < 0.01, N.S., Non-significant.

We initially assessed four variables to improve lipid production in *M. guilliermondii* BI281A in independent experiments (univariate approach). First, we analyzed the cultivation time using medium A (glucose) to establish the growth curve and maximum time of lipid production (Figure 2). Secondly, we analyzed different temperatures at 72 h of cell growth (Figure 3A). We found statistical differences for biomass and lipid production (Figure 3B, ANOVA, *P* < 0.05). The lowest biomass and lipid production were obtained at 20°C, compared to other temperatures (*P* < 0.05). However, there were no statistical differences between lipid production at 26, 28, 30, or 37°C (*P*-values between 0.1167 and 0.7391), and as temperatures between 25 and 28°C demand less energy input, we chose 26°C for the following experiments.

In the following step, we assessed different carbon and nitrogen sources to evaluate the ability of *M. guilliermondii* BI281A to assimilate, grow, and redirect the nutritional source to lipid production. For this purpose, we changed the composition of A-medium for each carbon or nitrogen source to be tested. We found that glycerol produced higher biomass than glucose (Figure 3C, *P* < 0.05), but we did not find significant differences in lipid production (*P* = 0.6290, Figure 3D). When we tested nitrogen sources, we found a higher biomass production (Figure 3E, *P* < 0.05) with peptone and tryptone, and lower production using malt extract and NH_4_NO_3_ when compared to (NH_4_)_2_SO_4_ (*P* < 0.05). However, no statistical differences in lipid production were found when peptone (Figure 3F, *P* = 0.6622), tryptone (*P* > 0.99), or NH_4_NO_3_ (*P* = 0.8382) were compared to ammonium sulfate. Peptone and tryptone are rich and variable sources of protein and amino acids for growing microorganisms (Gray et al., 2008), but their stoichiometric nitrogen composition cannot be controlled. For this reason, we decided to use ammonium sulfate as nitrogen source in the following experiments.

In order to validate the results using the RFU, we determined the lipid yield and productivity gravimetrically (Table 2). We found a strong correlation between the lipid production obtained from fluorescence measurements (in mg/L) and the lipid yield obtained gravimetrically (in g/L) (*R*^2^ = 0.9958), validating the measures using fluorescence (data not shown). In all treatments, *M. guilliermondii* BI281A proved to be a lipid producer (lipid content >37–52% w/w cell dry weight), except when malt extract and NH_4_NO_3_ were used as nitrogen sources. Moreover, the lipid productivity was highest using glucose in 24 h (Table 2). However, when we compared all the productivities at the same time (96 h), the values were similar (glucose = 0.0055 g/g/h; glycerol = 0.0036 g/g/h).

**Table 2:** *Meyerozyma guilliermondii* BI281 growth at six different combinations of carbon or nitrogen sources.

**Carbon/Nitrogen source**	**Time (h)**	**X (g L^−1^)**	**L (g L^−1^)**	**Lmax (%)**	**Ymax L X^−1^ t^−1^ (g/g/h)**
Glucose/(NH_4_)_2_SO_4_	24	1.9953 ± 0.1275	1.5247 ± 0.2659	76.41	0.0318
	48	2.2380 ± 0.2008	1.4770 ± 0.2984	65.99	0.0137
	72	2.4067 ± 0.2299	1.0580 ± 0.6633	43.96	0.0061
	96	2.5007 ± 0.4146	1.3100 ± 0.2348	52.39	0.0055
Glycerol/(NH_4_)_2_SO_4_	24	2.3733 ± 0.0811	0.6266 ± 0.0277	26.43	0.0110
	38	3.6944 ± 0.1195	0.6353 ± 0.0810	17.41	0.0046
	48	4.2649 ± 0.2804	0.7688 ± 0.0539	18.76	0.0039
	72	5.0889 ± 0.4714	0.7590 ± 0.0984	14.15	0.0020
	96	3.5633 ± 0.3523	1.2362 ± 0.0387	34.97	0.0036
	120	5.5484 ± 1.1415	1.3450 ± 0.3687	22.10	0.0018
Glucose/Malt extract	96	0.9747 ± 0.1897	0.5400^*^	ND	0.0077^*^
Glucose/NH_4_NO_3_	96	0.9473 ± 0.1099	0.1393 ± 0.0133	14.71	0.0020 ± 0.0017
Glucose/Peptone	96	5.6580 ± 0.2910	2.6613 ± 0.1453	47.04	0.0065 ± 0.0069
Glucose/Tryptone	96	5.9173 ± 0.2453	2.8953 ± 0.2404	48.93	0.0068 ± 0.0136

*Biomass (X), lipid production (L), lipid productivity (Ymax L/X/t) and maximum concentration of cellular lipids (Lmax) (%, according to total cell dry weight) are represented. All culture media were supplemented with 0.1% KH_2_PO_4_ and 0.05% MgCl_2_6H_2_O. The culture conditions were 150 rpm, 26°C for 96 h for glucose and 120 h for glycerol*.

* *Value without triplicate. For this reason, the estimation of Lmax is not shown (ND)*.

### Optimization of lipid production by *M. guilliermondii* Bl281A

After defining the best combination of carbon and nitrogen sources and the temperature of incubation, we designed a multivariate experimental design (CCD) to improve the lipid production in *M. guilliermondii* BI281A. Table 1 shows the maximum and minimum levels of variables chosen for trials in the CCD. The experimental responses are presented in Table 3, showing considerable variations in the amount of lipid production depending on the three independent variables tested. The response variable, lipid production expressed in RFU, was transformed to mg/L according to the calibration curve and varied from 10.13 to 198.79 mg/L. As shown in Table 3, there was a correspondence between observed and predicted values of the above parameters.

**Table 3:** Experimental design to improve *M. guilliermondii* BI281 lipid content, and comparison of observed data, data predicted by the model and residues.

**Run**	**Time (h)**	**C/N ratio**	**Stirring (rpm)**	**Lipid content (mg/L)**		
				**Observed value**	**Predicted value**	**Residues**
4 (c)	72	100:1	150	100.4719	126.4801	−26.0082
2	72	100:1	276	184.5952	162.8444	21.7508
12	96	50:1	225	57.2506	57.1579	0.0927
14	96	150:1	225	192.5581	185.1536	7.4045
5 (c)	72	100:1	150	198.7951	126.4801	72.3150
6 (c)	72	100:1	150	106.1470	126.4801	−20.3331
15	72	100:1	24	142.7192	90.1158	52.6034
8	72	16:1	150	16.8804	5.4506	11.4298
10	48	50:1	75	55.7712	113.4219	−57.6507
1	48	150:1	75	111.6914	119.8489	−8.1576
13	48	150:1	225	192.5581	190.2689	2.2892
16	32	100:1	150	110.2632	134.2331	−23.9699
9	112	100:1	150	14.0873	69.6156	−55.5284
11	96	50:1	75	72.8236	40.9963	31.8273
3	96	150:1	75	10.1330	18.1979	−8.0648

**Figure 4:**
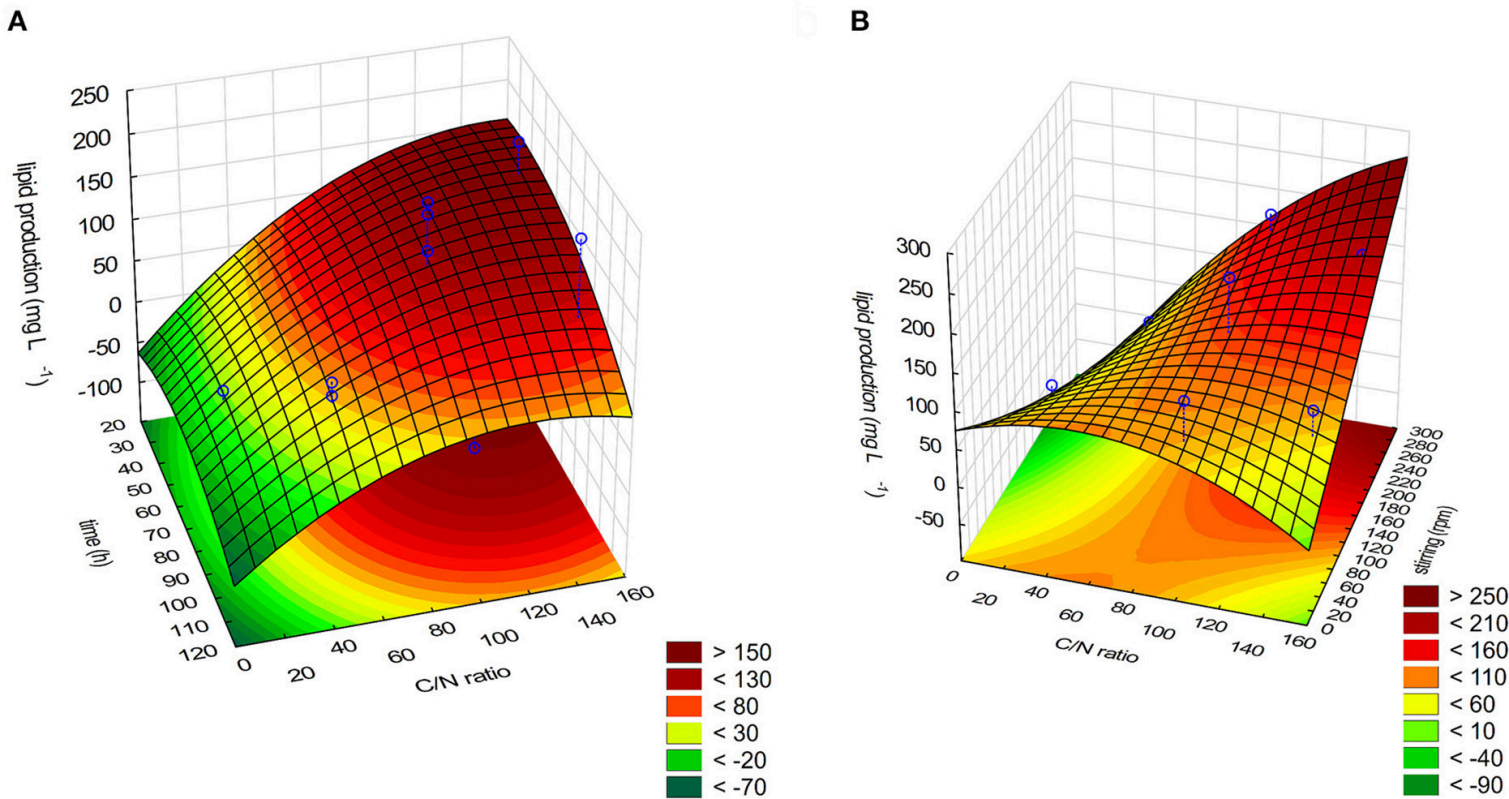
**(A)** Response surface plot for the effect on time cultivation, C/N ratio and their mutual effect on the lipid production in *M. guilliermondii* BI281A. **(B)** Response surface plot for the effect on C/N ratio, stirring (rpm) and their interaction on the lipid production for *M. guilliermondii* BI281A.

The experimental results of the CCD were fitted with a second-order polynomial regression. The values of the regression coefficients were calculated, and the fitted equation for predicting lipid production was simplified as Equation (2):

(2) $$\text{Z}=154.819-0.015{\text{X}}_{1}^{2}+1.433{\text{X}}_{2}+0.010{\text{X}}_{3}{\text{X}}_{2}-252.307$$

where Z is the lipid production (in mg/L) and X_1_, X_2_, and X_3_ are the variables expressed in Table 1. Fitting the above-optimized concentrations of the variables to Equation (2), the model predicted that the lipid production could reach 264.7 mg/L. The MANOVA for lipid production (in mg/L) showed *R*^2^ = 0.8306 (Table 4). To confirm the model adequacy for predicting maximum lipid production, three additional experiments using the optimum medium composition and cultivation conditions were performed. Biological triplicate experiments (with technical replicate) yielded an average highest lipid production of 169.51 ± 18.13 mg/L. A Chi-square test between the predicted (126.48 mg/L) and experimental results showed a higher value in the experimental results (Chi-Square = 100.8339, *df* = 5, *P* < 0.05), suggesting it is possible that *M. guilliermondii* BI281A can produce higher values than expected, possibly because of adaptation of the strain to the optimized medium (sub-culturing effect). As shown in Table 4, C/N ratio (positive linear effect), time of incubation (negative quadratic effect), and interaction of stirring and C/N ratio (positive effect) were statistically significant (*P* < 0.05) on the model to determine lipid production. Therefore, the three-dimensional plot of the combined different C/N ratios by time of cultivation (Figure 4A), and C/N ratio by stirring (Figure 4B) centered in the optimal conditions were constructed with the model. Higher C/N ratios and higher agitation speed positively affect the lipid production. However, if the C/N ratio is lower than 80:1, the lipid production tends to decrease.

**Table 4:** Analysis of variance for the fitted quadratic polynomial model for optimization of cellular lipid production (*P* < 0.05).

**Factor**	**SS**	**df**	**MS**	***F***	***P***
Time (hours) (Q)	11500.52	1	11500.52	7.57393	0.028419
C/N ratio (L)	25209.70	1	25209.70	16.60243	0.004722
C/N ratio (Q)	4653.90	1	4653.90	3.06494	0.123468
Stirring (rpm) (L)	104.96	1	104.96	0.06912	0.800186
Time by C/N ratio	5893.70	1	5893.70	3.88143	0.089473
Time by stirring	2386.91	1	2386.91	1.57196	0.250172
C/N ratio by stirring	12031.24	1	12031.24	7.92345	0.025964
Error	10629.04	7	1518.43		
Total SS	62746.97	14			

*R^2^ = 0.8306*.

*Q, quadratic effect; L, linear effect; SS, sum of squares; df, degrees of freedom; MS, mean squares*.

To validate the experimental design, we ran the growth kinetics of *M. guilliermondii* BI281A using the C/N ration and stirring in optimized conditions. The kinetics of BI281A changed slightly depending on the carbon source. The growth kinetics in glucose (Figure 2) showed that cells reached the stationary phase in 24 h. On the other hand, cells grown in glycerol reached the stationary phase in 48 h with a maximum lipid production of 108.65 ± 30 mg/L in 120 h. Our results suggested that cells grown in glycerol 15 % (v/v) (A-glycerol medium) for 120 h was the optimized point of lipid production based on the proposed model. The production was three times higher than non-optimized conditions using A medium (glucose 10% v/v, 72 h) (108.65 > 43.96 mg/L).

**Figure 5:**
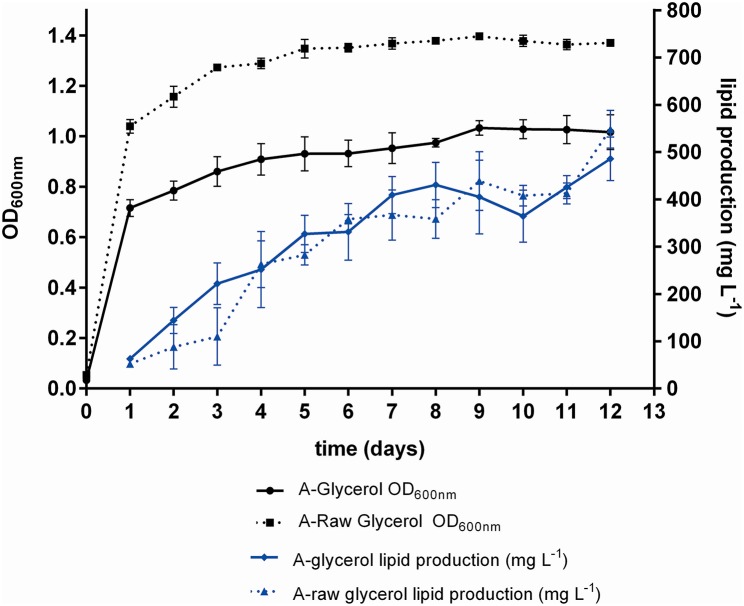
Growth curve (OD600 nm) and kinetics of lipid production (mg/L) of *M. guilliermondii* BI281A. The solid lines indicate pure glycerol and dotted lines indicate raw glycerol. The experiments were measured in biological triplicate (average ± standard deviation) for 12 days. Samples in 5 and 12th day were taken for lipid extraction, transesterification and profiling.

The FAME lipid profiles of *M. guilliermondii* BI281A were assessed in medium A-glycerol and A-raw glycerol, with results shown in Table 5. Also, we measured biomass and lipid production in 12 days of grown (Figure 5). The main types of FAME were monounsaturated fatty acids (MUFA), varying in a range of 35–73% of total FAME, and saturated fatty acids (SFA), representing around 21–51% of total FAME. A low percentage of poly-unsaturated fatty acids were produced (4–15%). The main fatty acid methyl ester found in 5 days of growth was oleic acid (C18:1n9, 48.76%), which is completely converted to elaidic acid (C18:1n9 trans) in 12 days (53.70%). Palmitic, palmitoleic, and linolelaidic acids were present in all samples and replicates.

**Table 5:** Fatty acid methyl esters profile of lipids produced by *M. guilliermondii* BI281 grown in pure glycerol (5 and 12 days) or raw glycerol (12 days).

	**Pure glycerol 5 days**	**Pure glycerol 12 days**	**Raw glycerol 12 days**
Lauric acid (C12:0)	8.0685^**^	N.D.	N.D.
Palmitic acid (C16:0)	25.7457^**^	24.3570 ± 10.31	15.2358 ± 1.17
Palmitoleic acid (C16:1)	N.D.^*^	7.8320 ± 1.33	7.4302 ± 0.01
cis-10-Heptadecenoic acid (C17:1)	N.D.	N.D.	6.4178 ± 0.74
Stearic acid (C:18:0)	34.1755 ± 10.23	13.7788 ± 3.03	6.4160 ± 1.37
Elaidic acid (C18:1n9 *trans*)	N.D.	53.7056 ± 8.29	59.84 ± 0.68
Oleic acid (C18:1n9 *cis*)	48.7601 ± 13.91	N.D.	N.D.
Linolelaidic acid (C18:2n6c)	8.1370^**^	11.0562 ± 9.56	4.6632 ± 0.15
γ-Linolenic acid (C18:3n3)	4.2056^**^	N.D.	N.D.
SFA^*^	51.29	34.44	21.65
MUFA^*^	35.11	55.57	73.68
PUFA^*^	15.08	9.98	4.66

*Experiments were done in triplicate and values are presented in percentage (% average ± standard deviation)*.

* *SFA, Saturated fatty acid; MUFA, Mono unsaturated fatty acid; PUFA, Poly unsaturated fatty acid; N.D., Non-detected*.

** *Fatty acid methyl ester was detected only in one sample*.

## Discussion

Brazilian biomes represent important hotspots of species richness on the planet, with a microbial biodiversity still largely unknown or unexplored for biotechnological purposes. Yeasts are promising microbial resources to produce various compounds, including lipids, which are of great interest for biodiesel and food industries. In this sense, some Brazilian ecosystems were assessed for the isolation of lipid-producer yeasts, such as Cerrado, Amazon Forest, and Pantanal (Duarte et al., 2013). Vieira et al. (2014) assessed wild yeasts to produce microbial lipids using Brazilian molasses as raw material to accumulate fatty acids. In our group, we previously analyzed yeasts isolated from artisanal cheese as lipid producers for biodiesel or feed supplement (Poli et al., 2013, 2014a; Mattanna et al., 2014a,b), or as model for lipid metabolism studies (Rosa et al., 2014, 2015). In the present study, we assessed a Brazilian collection of yeasts isolated from the phylloplane of bromeliads and macrophytes, and selected the oleaginous yeast *M. guilliermondii* BI281A.

*Meyerozyma guilliermondii* (formerly *Pichia guilliermondii*), an ascomycetic yeast, has been previously reported as oleaginous by Wang et al. (2012). Kurtzman et al. (2015) stated that an overview of closely related species of well reported oleaginous yeasts may have a predictive value for the prospection of biotechnologically relevant new strains. The genus *Meyerozyma* includes other reported oleaginous yeast species, such as *M. caribbica* (Polburee et al., 2015), and we showed that *M. guilliermondii* BI281A is also oleaginous. Our results were comparable with literature reporting oleaginous yeast belonging to the genus *Meyerozyma*. For example, Polburee et al. (2015) reported lipid content of 37.6% (w/w) for *M. caribbica* DMKU-RK258. Also, Wang et al. (2012) reported 60.6% (w/w) of total lipids for *M. guilliermondii* PCLA22 using inulin as carbon source and fed-batch fermentation.

Glycerol utilization for lipid accumulation is an interesting approach because *M. guilliermondii* BI281A grew better in glycerol than glucose, producing high yields of lipids, and there is currently a trend to use residual raw glycerol from the biodiesel industries as carbon source in bioprocesses (Thompson and He, 2006; de Souza et al., 2014; Spier et al., 2015; Tchakouteu et al., 2015). Our results suggested that there was no difference in lipid production between growth in pure glycerol or raw glycerol (Figure 5), however, the percentage of MUFA (relative to total fatty acid methyl esters) was higher in raw glycerol (after 12 days), suggesting raw glycerol as a good residual feedstock for lipid accumulation. The lipid profile of *M. guilliermondii* is suitable for biodiesel production, according to the criteria exposed by Sitepu et al. (2014): production of desired fatty acids (oleic, palmitic, and stearic acids) and identification of appropriate culture conditions.

According to Ageitos et al. (2011), the economic feasibility of the fermentation process to produce microbial lipids is determined by the cost of the raw materials in addition to the fermentation process itself. Therefore, minimizing the cost of carbon sources is an important factor that industry considers. A variety of chemical and lignocellulosic sources have been used as feedstock for lipid accumulation, including glucose (e.g., Ratledge, 2004; Li et al., 2007), fatty acids (Papanikolaou et al., 2001; Ratledge, 2004), glycerol (Pan et al., 2009), general wastewaters (Poli et al., 2014b), and wastewaters of animal fat treatment (Papanikolaou et al., 2001). With the ever-growing production of biodiesel in recent years, the annual amount of produced raw glycerol tends to increase in time (Duarte et al., 2013), consequently, cultivation of oleaginous yeasts in glycerol-based media is of great interest (Polburee et al., 2015).

In conclusion, the applied high-throughput screening methodology allowed a rapid estimation of the cellular lipid production in yeasts, being useful for the screening of oleaginous yeast cells and for the optimization of culture conditions to improve lipid production. *M. guilliermondii* BI281A was reported as an oleaginous yeast. It has potential to accumulate lipids in glucose or in glycerol as carbon sources, producing up to 52.38 or 34.97 % of total lipids (relative to dry weight). Also, the lipid profile showed 56 to 74% of MUFA in relation to the biomass dry weight in pure or raw glycerol, respectively. We suggest the oil produced by *M. guilliermondii* BI281A as a new potential biodiesel feedstock.

## Author contributions

Conceived and designed the experiments: MR, PV, PR, and ML. Performed the experiments: MR, VJ, and MF. Analyzed the data: MR, VJ, DV, MA, VR, JH, and PV; Wrote the manuscript: MR and PV.

### Conflict of interest statement

The authors declare that the research was conducted in the absence of any commercial or financial relationships that could be construed as a potential conflict of interest.
