# Supplementary material for: The Oleaginous Yeast Meyerozyma guilliermondii BI281A as a New Potential Biodiesel Feedstock: Selection and Lipid Production Optimization
Source: Front Microbiol. 2017 Sep 22;8:1776. doi: 10.3389/fmicb.2017.01776 (PMC5614974; doi:10.3389/fmicb.2017.01776)
Supplement: Supplementary file 1 [file DataSheet1.DOCX]

Supplementary Material

The oleaginous yeast *Meyerozyma guilliermondii* BI281A as a new potential biodiesel feedstock: selection and lipid production optimization

Mauricio Ramírez-Castrillón^1,2,3^, Victoria P. Jaramillo-Garcia^1^, Priscila Dallé da Rosa^4^, Melissa Fontes Landell^5^, Duong Vu^6^, Mariana F. Fabricio^7^, Marco Antônio Záchia Ayub^7^, Vincent Robert^6^, João Antonio Pegas Henriques^1^, Patrícia Valente^2*^

*** Correspondence:** Patricia Valente da Silva [patricia.valente@ufrgs.br](mailto:patricia.valente@ufrgs.br)

# Supplementary Figures and Tables

## Supplementary Tables

**Table S1.** Strains tested in this work.

| **Species** | **Strain id** | **Reference** |
| --- | --- | --- |
| *Papiliotrema* sp*.* (formerly *Auriculibuller* sp.) | BEL90 | Unpublished |
| *Fibulobasidium sp.* | BI14 | Landell *et al.* 2006 |
| *Candida parapsilosis* | BI06 | Landell *et al.* 2006 |
| *Farysia itapuensis* (formerly *Farysizima itapuensis*) | BI109 | Inacio *et al.* 2008 |
| *Papiliotrema* sp. | BI111 | Landell *et al.* 2006 |
| *Papiliotrema flavescens* | BI144 | Landell *et al.* 2006 |
| *Candida vrieseae* | BI146 | *Landell et al. 2010* |
| Non identified | BI147 | Landell *et al.* 2006 |
| *Slooffia tsugae (*formerly *Sporobolomyces tsugae*) | BI173 | Landell *et al.* 2006 |
| *Cryptococcus* sp. nov. | BI195 | Landell *et al.* 2006 |
| *Sirobasidium intermedium* | BI198 | Landell *et al.* 2006 |
| *Occultifur externus* | BI206 | Landell *et al.* 2006 |
| *Sporobolomyces roseus* | BI212 | Landell *et al.* 2006 |
| Non-identified | BI227 | Landell *et al.* 2006 |
| *Papiliotrema flavescens* | BI231 | Landell *et al.* 2006 |
| *Farysia itapuensis* | BI232 | Inacio *et al.* 2008 |
| *Candida sp.* | BI233 | Landell *et al.* 2006 |
| *Sporobolomyces pararoseus* | BI234 | Landell *et al.* 2006 |
| *Sporobolomyces roseus* | BI245 | Landell *et al.* 2006 |
| *Papiliotrema flavescens* | BI276 | Landell *et al.* 2006 |
| *Farysia itapuensis* | BI277 | Inacio *et al.* 2008 |
| *Papiliotrema flavescens* | BI278 | Landell *et al.* 2006 |
| *Rhodotorula nothofagi* | BI280 | Landell *et al.* 2006 |
| *Meyerozyma guilliermondii* | BI281A | This work |
| *Papiliotrema flavescens* | BI282 | Landell *et al.* 2006 |
| *Farysia itapuensis* | BI286 | Inacio *et al.* 2008 |
| *Papiliotrema flavescens* | BI296 | Landell *et al.* 2006 |
| Non-identified | BI300 | Landell *et al.* 2006 |
| *Farysia itapuensis* | BI302 | Inacio *et al.* 2008 |
| *Papiliotrema leoncinii* | BI309 | Pagani *et al.* 2016 |
| *Slooffia tsugae* | BI316 | Landell *et al.* 2006 |
| *Hannaella kunmingensis* | BI320 | Landell *et al.* 2006 |
| Non-identified | BI396 | Landell *et al.* 2006 |
| Non-identified | BI67 | Landell *et al.* 2006 |
| *Priceomyces melissophilus* | BI81 | Landell *et al.* 2006 |
| *Sporobolomyces pararoseus* | BI89 | Landell *et al.* 2006 |
| *Papiliotrema sp.* | DEC07 | Unpublished |
| *Sterigmatomyces elviae* | DEC37 | Unpublished |
| Non-identified | DEC49 | Unpublished |
| *Cystobasidium sp.* | DEC79 | Unpublished |
| *Rhodotorula mucilaginosa* | DEC87 | Unpublished |
| *Candida parapsilosis* | EI01 | Landell *et al.* 2006 |
| *Taphrina* sp. nov. | EI05 | Landell *et al.* 2006 |
| *Saccharomyces cerevisiae* (negative control) | CBS1171 |  |
| *Yarrowia lipolytica* (positive control) | QU21 | Poli et al. 2013 |

## Supplementary Figures

**
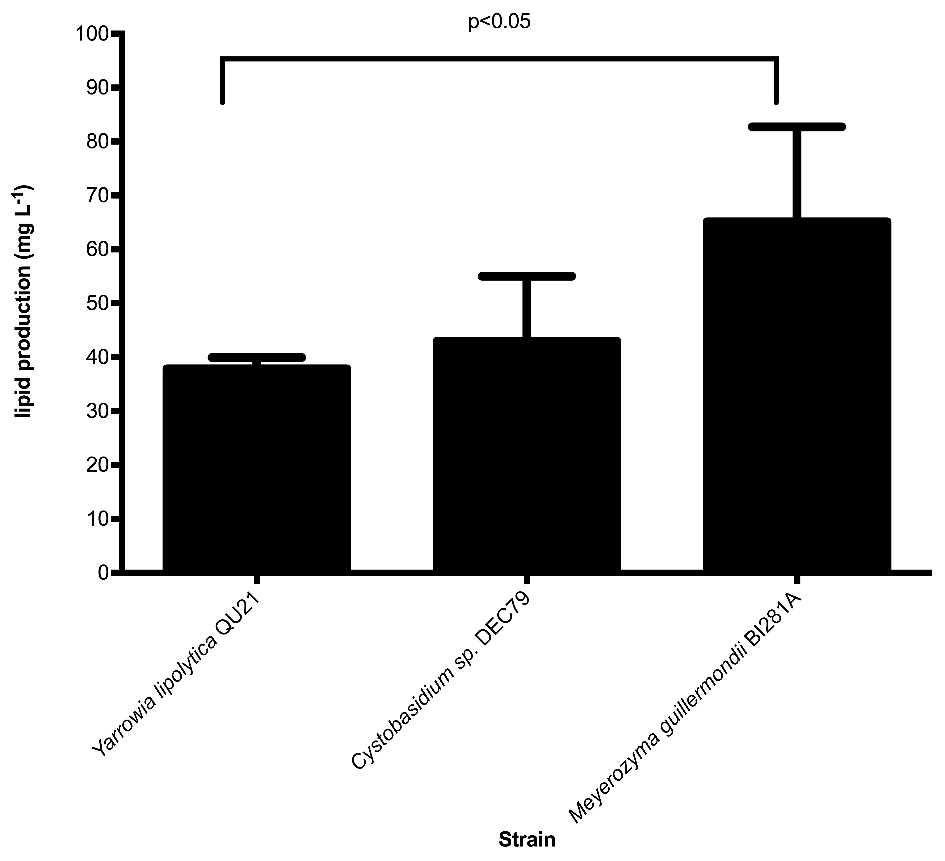
**

**Supplementary Figure S1.** Assessment of lipid content in *M. guilliermondii* BI281A and *Cystobasidium sp.* DEC79 as candidate oleaginous yeasts compared to *Y. lipolytica* QU21.

# References

Inácio, J., Landell, M., Valente, P., Wang, P., Mansson, J., Lachance, M.A., Rosa, C.A., Fonseca, A. (2008). *Farysizyma* gen. nov., an anamorphic genus in the Ustilaginales to accommodate three novel epiphytic basidiomycetous yeast species from America, Europe and Asia. *FEMS Yeast Res.,* 8: 499-508.

Landell, M.F., Mautone, J.N., Valente, P. (2006). Biodiversity of yeasts associated to bromeliads in Itapuã park, Viamão/RS. *Biociências*, 14(2): 144-149.

Landell, M.F., Billodre, R., Ramos, J.P., Leoncini, O., Vainstein, M.H., Valente, P. (2010). *Candida aechmeae* sp. nov. and *Candida vrieseae* sp. nov., novel yeast species isolated from the phylloplane of bromeliads in Southern Brazil. *Int. J. Syst. Evol. Microbiol.,* 60(1): 244-248.

Pagani, D.M., Brandão, L.R., Santos, A.R.O., Felix, C.R., Ramos, J.P., Broetto, L., Scorzetti G., Fell J., Rosa C.A., Valente P., Landell, M.F. (2016). *Papiliotrema leoncinii* sp. nov. and *Papiliotrema miconiae* sp. nov., two tremellaceous yeast species from Brazil. *Int. J. Syst. Evol. Microbiol.* 66(4), 1799-1806.

Poli, J.S., Rosa, P.D., Senter, L., Mendes, S.D.C., Ramirez-Castrillon, M., Vainstein, M.H., Valente, P. (2013). Fatty acid methyl esters produced by oleaginous yeast *Yarrowia lipolytica* QU21: an alternative for vegetable oils. *R. Bras. Bioci.,* 11(2): 203-208.
